# “Mitochondrial CA‐V inhibition mitigates Alzheimer’s Disease progression via modulation of neurovascular inflammation and microglia function”

**DOI:** 10.1002/alz70861_108889

**Published:** 2025-12-23

**Authors:** Nicole L Lemon, Elisa Canepa, Tina Hirt, Maryam H. Abyaneh, Marc A Ilies, Silvia Fossati

**Affiliations:** ^1^ Alzheimer's Center at Temple, Lewis Katz School of Medicine, Philadelphia, PA USA; ^2^ Temple University School of Pharmacy, Philadelphia, PA USA

## Abstract

**Background:**

The neurovascular unit is known to become dysfunctional in Alzheimer’s Disease (AD). Amyloidosis disrupts the function of neurovascular cells via extrinsic and intrinsic apoptosis, and induction of blood brain barrier (BBB) permeability. Findings in our lab demonstrated that pan‐Carbonic Anhydrase (CA) inhibitors prevent mitochondria‐mediated apoptosis and inflammatory activation in multiple neurovascular cell types. Therefore, we hypothesized that the mitochondrial CA isoforms, CA‐VA and ‐VB mediate Aβ‐induced toxicity. Importantly, mitochondrial isoform CA‐VB is increased in models of amyloidosis. We have recently demonstrated that selective CA‐V inhibition and ‐VB KO are effective to rescue cerebrovascular cell death and BBB dysfunction. Here, we hypothesized that CA‐V inhibition would prevent apoptosis in 3xTG mice brains. Furthermore, we hypothesized that CA‐V inhibition would ameliorate neuroinflammation and microglia function, preventing the progression of AD pathology and cognitive impairment.

**Methods:**

3xTG AD mice were treated with a CA‐V inhibitor at 20mg/kg in their diet from 6‐16 months of age. We evaluated caspase‐3 activation, GFAP and IBA1 through immunohistochemistry. To measure the extent of amyloid and tau accumulation we extracted soluble and insoluble fractions from the brain and quantified the expected species using ELISA sandwich assay. To assess microglia function in vitro we used the human microglia cell line (HMC3) WT and CA‐VB KO, in the presence or absence of the same CA‐V inhibitor.

**Results:**

CA‐V inhibition prevented apoptosis and gliosis in the brains of 3xTG mice. Additionally, we observed mitigation of insoluble amyloid and tau accumulation. We also observed that, when HMC3 cells were treated with amyloid in the presence of CA‐V inhibitor there was an enhancement in lysosomal maker CD68. Strikingly, in CA‐VB KO cells, the CA‐V inhibitor did not have the same effect, implying a CA‐VB specific mechanism.

**Conclusions:**

These findings improve our understanding of the function of CA‐V in the development of AD pathology and highlight the potential of CA‐VB inhibition to improve microglial clearance and reduce brain neuroinflammation. Overall, we demonstrate that mitochondrial CA‐V is emerging as a promising target against AD and other cerebrovascular indications.

